# Chitosan nanoparticle encapsulated pentoxifylline improves renal protection and reduces oxidative stress in amikacin induced nephrotoxicity

**DOI:** 10.1186/s11671-026-04515-8

**Published:** 2026-04-18

**Authors:** Nada Moustafa, Mona B. Abd El-latif, Alyaa Farid

**Affiliations:** 1 Biotechnology Department, Faculty of Science, Cairo University, Giza, Egypt; 2 Environmental Research Department, Theodor Bilharz Research Institute, Giza, Egypt

**Keywords:** Pentoxifylline, Chitosan nanoparticles, Nephrotoxicity

## Abstract

Amikacin (AMK)-induced nephrotoxicity remains a clinical challenge, necessitating innovative therapeutic strategies. This study developed pentoxifylline-loaded chitosan nanoparticles (PTX-CSNPs) to enhance PTX’s renoprotective effects while minimizing its side effects. PTX-CSNPs were synthesized via ionic gelation, exhibiting high entrapment efficiency (89.53%) and sustained release (87% at 48 h). Characterization revealed spherical nanoparticles (30-50 nm by TEM and 79.05 nm by DLS) with positive zeta potential (+ 32.77 mV), confirmed by FTIR and XRD analyses. In vitro, PTX-CSNPs demonstrated superior antioxidant (98.73% DPPH scavenging), anti-inflammatory (76.46% RBCs membrane stabilization), and anticoagulant activity compared to free PTX. In vivo, AMK-induced nephrotoxic rats treated with PTX-CSNPs showed normalization of renal function biomarkers (BUN, creatinine, uric acid) and oxidative stress markers (MDA, SOD, GSH), alongside reduced inflammatory markers levels outperforming free PTX or CSNPs alone. Histopathology revealed preserved glomerular and tubular architecture. The nanoformulation’s efficacy was attributed to chitosan-mediated targeted delivery and PTX’s anti-inflammatory/antioxidant synergy. These findings highlighted PTX-CSNPs as a promising nanotherapeutic for drug-induced kidney injury, offering enhanced bioavailability and reduced systemic toxicity.

**Graphical abstract:**

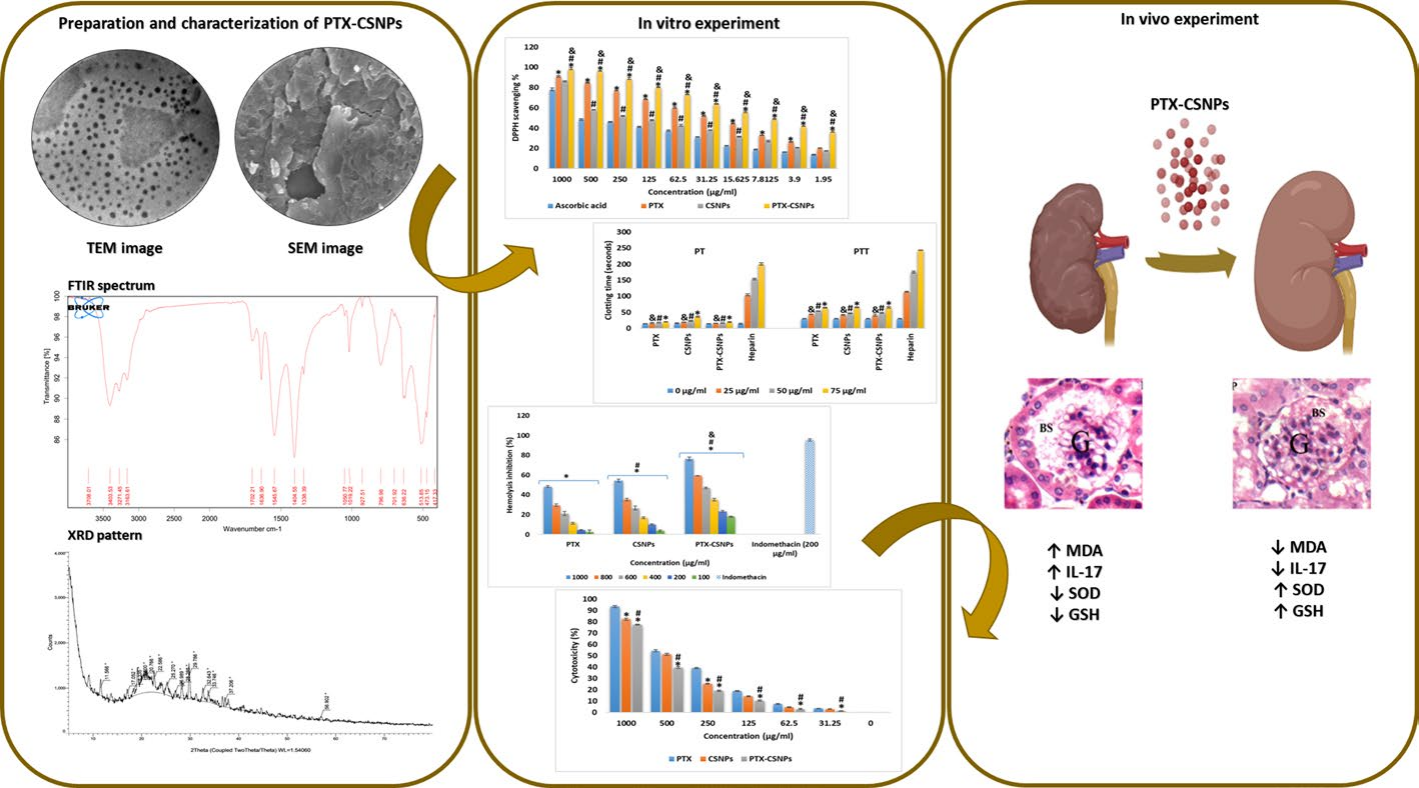

## Highlights

PTX-loaded chitosan nanoparticles (PTX-CSNPs) demonstrated superior antioxidant and anti-inflammatory activity compared to free PTX.

PTX-CSNPs exhibited high entrapment efficiency and sustained release, addressing PTX’s limitations of rapid clearance and dose-dependent side effects while enabling targeted renal therapy.

In amikacin-induced nephrotoxicity, PTX-CSNPs normalized kidney function biomarkers and oxidative stress markers.

## List of chemicals, kits and apparatus

- 1,1-Diphenyl-2-picrylhydrazyl (DPPH) (Sigma-Aldrich, USA)
- Amikacin (AMK) (Sigma-Aldrich, USA)
- BioTek Synergy H1 Microplate Reader (BioTek, USA)
- Bruker D8 Advance X-ray Diffractometer (Bruker, USA)
- Bruker Tensor II FTIR Spectrometer (Bruker, Germany)
- Chitosan (medium molecular weight, ≥75% deacetylated) (Sigma-Aldrich, USA)
- Creatinine Assay Kit (ab65340) (Abcam, USA)
- Glutathione (GSH) Assay Kit (MBS267424) (MyBioSource, USA)
- Hitachi SU3500 SEM (Hitachi, Japan)
- IL-17 ELISA Kit (E-EL-M0047) (Elabscience, USA)
- JEOL JEM-1400Flash TEM (JEOL, Japan)
- Malondialdehyde (MDA) Assay Kit (MBS741034) (MyBioSource, USA)
- Malvern Zetasizer Nano ZS90 (Malvern Panalytical, UK)
- Pentoxifylline (PTX) (Sigma-Aldrich, USA)
- Shimadzu UV-1800 Spectrophotometer (Shimadzu, Japan)
- Sodium Tripolyphosphate (TPP) (Sigma-Aldrich, USA)
- Superoxide Dismutase (SOD) Assay Kit (MBS2707323) (MyBioSource, USA)
- Sysmex CA-560 Coagulation Analyzer (Sysmex, Japan)
- Urea Assay Kit (ab83362) (Abcam, USA)
- Uric Acid Assay Kit (ab65344) (Abcam, USA)

## Introduction

The kidneys are vital organs responsible for maintaining systemic homeostasis through critical functions such as detoxification, regulation of extracellular fluid balance, and excretion of metabolic waste and xenobiotics [1]. Due to their high perfusion rate and excretory role, the kidneys are particularly vulnerable to injury from exogenous toxins, including therapeutic drugs [2]. Drug-induced nephrotoxicity, characterized by impaired renal excretion and filtration, accounts for approximately 20% of acute kidney injury cases, with incidence rates that reach 66% in elderly populations due to polypharmacy and age-related decline in renal function [3]. Notably, the clinical utility of many chemotherapeutic agents is limited by their dose-dependent nephrotoxic effects [4]. Current diagnostic methods rely on blood biomarkers like serum creatinine, blood urea nitrogen (BUN), and glomerular filtration rate (GFR). However, these parameters only detect functional impairment after significant renal damage has occurred, highlighting the need for more sensitive early biomarkers [5].

Aminoglycosides represent a critically important class of antibiotics with broad-spectrum activity against Gram-negative and some Gram-positive bacterial infections [6]. Beyond their antimicrobial applications, these compounds have demonstrated therapeutic potential for genetic disorders and Meniere’s disease through nonsense mutation suppression [7] and are being investigated as potential HIV replication inhibitors [8]. Among aminoglycosides, amikacin (AMK) has emerged as particularly valuable due to its resistance to most aminoglycoside-modifying enzymes, making it effective against otherwise resistant infections [9]. Its pharmacokinetic profile shows rapid distribution with peak serum concentrations occurring 30–60 min post intravenous administration [10]. Current clinical practice favors once-daily dosing regimens [11, 12], which optimize therapeutic efficacy while attempting to mitigate the characteristic nephro- and ototoxicity associated with this drug class [13, 14]. This balance between clinical benefit and toxicity risk necessitates careful consideration of dosing strategies, particularly in vulnerable populations.

Given amikacin’s well-documented nephrotoxicity, which occurs in up to 20–30% of treated patients [15], the concurrent use of nephroprotective agents is often clinically warranted. This is particularly crucial for high-risk populations, including the elderly, critically ill patients, and those receiving prolonged or high-dose regimens. Protective strategies may include hydration protocols, antioxidants like N-acetylcysteine (NAC), or renoprotective drugs, which has demonstrated efficacy in mitigating aminoglycoside-induced renal injury through its anti-inflammatory and antioxidant properties [16]. Such adjunctive therapies aim to preserve renal function while maintaining amikacin’s antimicrobial efficacy, especially in cases where alternative antibiotics are limited by resistance patterns.

Pentoxifylline (PTX), a non-specific phosphodiesterase inhibitor, has emerged as a promising adjunct therapy for chronic kidney disease (CKD) due to its multifaceted mechanisms of action. Originally used for peripheral vascular diseases due to its hemorheological effects such as reducing blood viscosity, improving erythrocyte deformability, and inhibiting platelet aggregation [17, 18]. PTX has since been recognized for its anti-inflammatory, antifibrotic, and renoprotective properties [19]. It suppresses pro-inflammatory cytokines, including tumor necrosis factor-alpha (TNF-α), interleukin-6 (IL-6), and interferon-gamma (IFN-γ), by inhibiting their gene transcription and mRNA accumulation [20, 21]. Additionally, PTX reduces oxidative stress and extracellular matrix deposition, key contributors to CKD progression [22–24]. Clinical studies and meta-analyses have demonstrated its efficacy in reducing proteinuria in diabetic nephropathy [23, 25, 26] and improving anemia in CKD patients by counteracting inflammation-induced erythropoietin resistance [27, 28]. Given its ability to modulate multiple pathways involved in CKD pathogenesis, PTX represents a valuable therapeutic option, particularly for patients with inflammatory or diabetic kidney disease [29].

PTX is generally well-tolerated, but some patients experience adverse effects, primarily gastrointestinal disturbances such as nausea, vomiting, dyspepsia, and bloating [30]. Cardiovascular effects, though less common, may include flushing, dizziness, hypotension, tachycardia, and dyspnoea. Rare but serious reactions include bleeding risk (due to its antiplatelet effects), allergic skin reactions, and angioedema [31]. Central nervous system (CNS) side effects, such as headache, dizziness, and agitation, have also been reported [32]. Due to its vasodilatory properties, caution is advised in patients with low blood pressure or bleeding disorders [33]. While most side effects are mild and dose-dependent, severe reactions warrant discontinuation and medical evaluation.

Although PTX has therapeutic potential in chronic inflammatory and vascular diseases, but its clinical use is limited by poor bioavailability, rapid clearance, and dose-dependent side effects. Encapsulating PTX in chitosan nanoparticles (CSNPs) could enhance its delivery by improving drug stability, prolonging circulation time, and enabling targeted release [34]. Chitosan, a biocompatible polymer, enhances mucosal adhesion and cellular uptake, potentially reducing systemic side effects while increasing drug accumulation at the disease sites (e.g., inflamed kidneys) [35]. In this study, CS NPs were selected as a delivery vehicle based on their well-documented propensity for renal accumulation, attributed to their positive surface charge and affinity for negatively charged glomerular and tubular membranes [36, 37]. This property was hypothesized to enhance renal drug availability and improve therapeutic outcomes compared to free PTX. Nanoparticle formulations may also allow lower dosing frequencies, minimize adverse effects while maintain efficacy. Such an approach could optimize PTX’s hemorheological and anti-inflammatory benefits for conditions like chronic kidney disease or peripheral artery disease.

## Materials and methods

### Preparation of pentoxifylline-loaded chitosan nanoparticles (PTX-CSNPs)

PTX-CSNPs were prepared using the ionic gelation method, exploiting the electrostatic interaction between positively charged chitosan and negatively charged sodium tripolyphosphate (TPP). First, 20 mg of chitosan (medium molecular weight, ≥ 75% deacetylated, Sigma-Aldrich, USA) was dissolved in 10 mL of 1% (v/v) acetic acid under magnetic stirring (500 rpm, 25 °C, 2 h) to yield a 0.2% (w/v) solution, and the pH was adjusted to 4.8 with 1 M NaOH. Separately, 10 mg of PTX (Sigma-Aldrich, USA) was dissolved in 1 mL of deionized water, and 10 mg of TPP (Sigma-Aldrich, USA) was dissolved in 10 mL of deionized water to prepare a 0.1% (w/v) TPP solution. The PTX solution was added dropwise to the chitosan solution with continuous stirring (500 rpm, 30 min). Subsequently, the TPP solution was added dropwise at a 1:2 (TPP: Chitosan) volume ratio under increased stirring (600 rpm, 30 min) to induce ionic cross-linking and nanoparticles formation. The resulting suspension was centrifuged (15,000 rpm, 30 min, 4 °C) to pellet the PTX-CSNPs. The pellet was washed twice with distilled water and resuspended in 10 mL of ultrapure water. For stability studies, the nanoparticle suspension was mixed with 2% (w/v) mannitol as a cryoprotectant, frozen at − 80 °C for 24 h, and lyophilized to obtain a dry powder.

### Drug release study and entrapment efficiency analysis

The in vitro release profile of PTX from PTX-CSNPs was evaluated using a dialysis bag method. Briefly, 2 mL of PTX-CSNPs suspension (equivalent to 1 mg/mL PTX) was placed in a pre-soaked dialysis bag and immersed in 50 mL of phosphate-buffered saline (PBS, pH 7.4) containing 0.1% Tween 80 to maintain sink conditions. The system was agitated at 100 rpm and 37 °C in a thermostated water bath. At predetermined intervals (0.5, 1, 2, 4, 6, 8, 12, 24, and 48 h), 1 mL aliquots were withdrawn and replaced with fresh medium to maintain constant volume. The PTX concentration in the samples was quantified using UV-Vis spectrophotometry at 274 nm [38]. For entrapment efficiency (EE%), PTX-CSNPs were separated by ultracentrifugation (15,000 rpm, 30 min, 4 °C). The supernatant was analyzed for free PTX content and the EE% was calculated as: EE% = [(Total PTX - Free PTX) / Total PTX] x 100. All experiments were performed in triplicate, and data were expressed as mean ± SD.

### Characterization of PTX-CSNPs

The physicochemical properties of PTX-CSNPs were evaluated using advanced analytical techniques. FTIR spectra were acquired using a Bruker Tensor II spectrometer with Platinum ATR. Scans (*n* = 32) were collected from 400 to 4000 cm^−1^ at 4 cm^−1^ resolution. OPUS software v8.7 was used for ATR correction and baseline subtraction. X-ray diffraction (XRD) analysis was conducted using a Bruker D8 Advance Diffractometer (Billerica, MA, USA) with Cu-Kα radiation (λ = 1.54 Å) operated at 40 kV and 40 mA. Scans were performed over a 2θ range of 5–80° at a step size of 0.02° and a scanning rate of 2° min^−1^ to evaluate crystallinity.

For morphological characterization, scanning electron microscopy (SEM) images were acquired using a Hitachi SU3500 Variable Pressure SEM (Tokyo, Japan) at an accelerating voltage of 15 kV. Samples were sputter-coated with gold-palladium (10 nm thickness) using a Quorum Q150R ES sputter coater to enhance conductivity. Transmission electron microscopy (TEM) was performed on a JEOL JEM-1400Flash TEM (Tokyo, Japan) operated at 120 kV. Nanoparticle suspensions were drop-cast onto carbon-coated copper grids (300 mesh) and air-dried before imaging.

Particle size distribution and surface charge were determined via dynamic light scattering (DLS) and zeta potential measurements using a Malvern Zetasizer Nano ZS90 (Malvern, UK). Samples were diluted in filtered deionized water (0.22 μm pore size) and analyzed at 25 °C with a scattering angle of 173°. Each measurement consisted of three runs with 12 sub-runs to ensure statistical reliability. Prior to analysis, samples were equilibrated for 2 min and sonicated for 30s to minimize aggregation.

### In vitro characterization of PTX-CSNPs

#### Determination of the antioxidant activity [DPPH (1, 1-diphenyl-2-picryl hydrazyl) method]

The free radical scavenging activities of PTX, CSNPs, and PTX-CSNPs were evaluated using the DPPH (Sigma-Aldrich, USA) method, with ascorbic acid as a positive control. Serial dilutions (1.95–1000 µg/mL) of each sample were prepared in methanol and mixed with 1 mL of 0.1 mM DPPH solution (in methanol) in amber vials to prevent photodegradation. The reaction mixtures were vortexed for 30s and incubated in the dark at 25 °C for 30 min to allow radical scavenging. Absorbance was measured at 517 nm using a UV-Vis spectrophotometer (Shimadzu UV-1800, Japan), with methanol as a blank. The scavenging activity (%) was calculated as [(A_0_ − A_1_)/A_0_] × 100, where A_0_ and A_1_ represent the absorbance of the control and test samples, respectively. All experiments were performed in triplicate under low-light conditions to minimize DPPH degradation.

#### Determination of the anticoagulant activity (PT and PTT assay)

The anticoagulant potential of PTX, CSNPs, and PTX-CSNPs was assessed via prothrombin time (PT) and partial thromboplastin time (PTT) using fresh rat plasma. Briefly, 900 µL of citrated plasma was incubated with 100 µL of samples (25–75 µg/mL) or heparin (control) at 37 °C for 5 min in a Sysmex CA-560 coagulation analyzer (Japan). For PT, 200 µL of prewarmed thromboplastin reagent was added to initiate clotting. For PTT, plasma was first mixed with 100 µL of PTT reagent and incubated for 3 min, followed by 100 µL of 25 mM CaCl₂ to trigger coagulation. Clotting times (in seconds) were recorded automatically, with each concentration tested in triplicate.

#### Determination of the anti-inflammatory activity (membrane stabilizing assay)

The anti-inflammatory activity of PTX, CSNPs, and PTX-CSNPs was determined by inhibition of hypotonicity-induced hemolysis of rat red blood cells (RBCs). Fresh heparinized rat blood was centrifuged at 1500 × g for 20 min to isolate RBCs, which were washed three times with isotonic phosphate-buffered saline (PBS, pH 7.4) and resuspended to a 10% (v/v) suspension. Test samples (100–1000 µg/mL) or indomethacin (200 µg/mL, control) were mixed with 0.1 mL RBCs suspension and 5 mL of hypotonic PBS (0.25% NaCl) or isotonic PBS, followed by incubation at 37 °C for 1 h. After centrifugation (1500 × g, 10 min), hemoglobin release was quantified at 540 nm using a microplate reader (BioTek Synergy H1, USA). The percentage inhibition of hemolysis was calculated as [1 − (A_h_ − A_i_)/(A_n_ − A_i_)] × 100, where A_h_, A_i_, and Aₙ represent absorbances of hypotonic, isotonic, and negative control samples, respectively.

#### Cytotoxicity assessment (MTT assay)

The cytotoxicity of PTX, CSNPs, and PTX-CSNPs was evaluated against two human kidney cell lines: HEK293T (human embryonic kidney cells, ATCC^®^ CRL-3216™) and HK-2 (human proximal tubular epithelial cells, ATCC^®^ CRL-2190™) using the 3-(4,5-dimethylthiazol-2-yl)-2,5-diphenyltetrazolium bromide (MTT; Sigma-Aldrich, USA) assay. HEK293T cells were cultured in RPMI-1640 medium (Gibco, USA), while HK-2 cells were maintained in Keratinocyte-Serum Free Medium (K-SFM, Gibco, USA) supplemented with 5 ng/mL human recombinant EGF and 0.05 mg/mL bovine pituitary extract. Both media were supplemented with 10% fetal bovine serum (FBS, Gibco, USA) and 1% penicillin-streptomycin (Gibco, USA). Cells were seeded at a density of 1 × 10^4^ cells/well in 96-well plates and incubated for 24 h at 37 °C in a 5% CO_2_ humidified atmosphere to allow adherence. After incubation, cells were treated with sample suspensions at concentrations ranging from 31.25 to 1000 µg/mL. Following 24 h of treatment, 20 µL of MTT reagent (5 mg/mL in PBS) was added to each well and incubated for an additional 4 h at 37 °C. The formazan crystals formed were dissolved in 100 µL of dimethyl sulfoxide (DMSO, Sigma-Aldrich, USA), and the absorbance was measured at 560 nm. All experiments were performed in triplicate.

### Experimental design

All experimental procedures were conducted in accordance with the ARRIVE Guidelines 2.0 and the Guide for the Care and Use of Laboratory Animals (8th edition, 2011, National Academies Press, USA). The study protocol was approved by the Institutional Animal Care and Use Committee (CU-IACUC), Cairo University, Egypt (Approval No. CUIF6723). A priori power analysis (G*Power 3.1) determined that a minimum of 5 mice per group was required to achieve 80% statistical power with an α-level of 0.05, assuming a medium effect size (f = 0.25) based on preliminary data. Based on a priori power analysis, 25 male BALB/c mice (8–10 weeks old, 25–30 g) were obtained from the National Research Centre (Cairo, Egypt) and randomly allocated into five experimental groups (*n* = 5/group) using a computer-generated randomization table. Mice were housed in individually ventilated cages (IVCs) under standardized conditions of temperature: 22 ± 2 °C, humidity: 50 ± 2%, light/dark cycle: 12 h (lights on at 07:00), standard rodent chow and water ad libitum. After a 7 day acclimatization period, animals were divided into: group I (NC): healthy controls, group II (PC): kidney injury induced by amikacin (100 mg/kg/day i.p. for 10 days), group III (PTX): amikacin-induced kidney injured mice treated with PTX (50 mg/kg/day) [39–41], group IV (CSNPs): amikacin-induced kidney injured mice treated with CSNPs (50 mg/kg/day) [42] and group V (PTX-CSNPs): amikacin-induced kidney injured mice treated with PTX-CSNPs (50 mg/kg/day) [40, 41, 43, 44]. All treatments were administered for 28 days post-kidney injury induction via oral gavage (22G needle) between 09:00 and 11:00 to minimize circadian variability. Amikacin (Sigma-Aldrich, USA) was dissolved in saline and injected i.p. (100 mg/kg/day for 10 days) as described by Batoo et al. [45]. Clinical signs (weight loss, fur texture and activity) were monitored daily. Urine output was measured weekly using metabolic cages (Tecniplast, Italy). At the end of experiment, mice were anesthetized with sodium pentobarbital (50 mg/kg i.p.) and exsanguinated via cardiac puncture. Blood was centrifuged at 1000 × g for 10 min (4 °C) and serum was stored at − 80 °C. Kidneys were collected, fixed in 10% neutral buffered formalin (24 h) for histopathological examination or were homogenized in ice-cold Tris-HCl (10 mM, pH 7.4) for biochemical assays.

### Kidney function analysis

Renal function was assessed through serum biochemistry and direct measurement of glomerular filtration rate (GFR) via creatinine clearance. Serum concentrations of urea, creatinine, and uric acid were quantified using commercial assay kits (Urea: ab83362, Abcam, USA; Creatinine: ab65340, Abcam, USA; Uric Acid: ab65344, Abcam, USA) according to the manufacturer’s protocols. To determine creatinine clearance, mice were housed individually in metabolic cages for 24 h urine collection at the end of the treatment period. Urine volume was recorded, and urine creatinine concentration was measured using the same creatinine assay kit (ab65340, Abcam, USA). Creatinine clearance (GFR) was calculated using the standard formula:

$$ {\mathrm{Creatinine~clearance~(mL/day)}} = \frac{{\left[ {{\mathrm{Urine~creatinine~(mg/dL)}}} \right] \times {\mathrm{Urine~volume~(mL/day)}}}}{{\left[ {{\mathrm{Serum~creatinine~(mg/dL)}}} \right]}} $$

### Liver function analysis

To evaluate potential hepatotoxicity, serum levels of aspartate aminotransferase (AST) and alanine aminotransferase (ALT) were quantified at the experimental endpoint using commercially available colorimetric assay kits (AST: ab263882, Abcam, USA; ALT: ab282882, Abcam, USA) according to the manufacturer’s instructions.

### Oxidative stress and immunological analysis

Kidney homogenates were analyzed for malondialdehyde (MDA, MBS741034, MyBioSource, USA), superoxide dismutase (SOD. MBS2707323, MyBioSource, USA), and glutathione (GSH, MBS267424, MyBioSource, USA). Inflammatory markers (IL-17, TNF-α, IL-6 and CRP) were measured in kidney tissue homogenates by mice ELISA kits (E-EL-M0047, Elabscience, USA; and ab208348, ab222503 and ab222511, respectively; Abcam, UK). ELISA procedures followed the manufacturer’s guidelines and precautions.

### Histopathological examination and scoring

Kidney tissues were fixed in 10% neutral buffered formalin, embedded in paraffin, and sectioned at 4 μm thickness. Sections were stained with hematoxylin and eosin (H&E) for general morphology. Periodic Acid–Schiff (PAS) staining was performed to highlight carbohydrate-rich structures by oxidizing sections in 0.5% periodic acid for 10 min, treating with Schiff reagent for 20 min, and counterstaining with hematoxylin. Histopathological evaluation was performed by two independent blinded observers using light microscopy. For H&E-stained sections, a semiquantitative scoring system was applied to five parameters: glomerular size (0: normal, 1: small/congested, 2: atrophied/necrotic), Bowman’s space dilation (0: absent, 1: widened/dilated, 2: obliterated), tubular damage (0: absent, 1: moderate, 2: severe), inflammatory cell infiltrate (0: none, 1: moderate, 2: marked), and capillary dilation (0: absent, 1: moderate, 2: marked). The total histological injury score per animal was calculated as the sum of these five parameters (maximum score = 10). PAS-stained sections were evaluated using a complementary scoring system assessing tubular brush border loss (0–2), protein cast formation (0–3), glomerular basement membrane thickening (0–2), and tubular dilatation (0–3). The total PAS injury score per animal was calculated as the sum of these four parameters (maximum score = 10).

### Statistical analysis

Statistical analysis was performed using one-way ANOVA followed by Tukey’s post hoc test for multiple comparisons. Data were reported as mean ± standard deviation (SD), with *p* < 0.05 considered statistically significant. No animals were excluded, ensuring complete data sets (*n* = 5/group).

## Results

### Drug release kinetics and EE%

The in vitro release profile of PTX from PTX-CSNPs demonstrated a biphasic pattern, characterized by an initial burst release followed by a sustained drug release. Within the first half h, 15.67 ± 2.08% of PTX was released, increasing to 23.67 ± 1.52% at 1 h and 30.33 ± 1.53% at 2 h, suggesting rapid diffusion of surface-adsorbed drug molecules. The release rate peaked between 4 and 12 h, reaching 43.00 ± 3.0% (4 h), 53.33 ± 2.51% (6 h), 70.33 ± 2.08% (8 h) and 83.33 ± 2.52% (12 h), indicative of progressive chitosan matrix erosion. By 24 h, the cumulative release reached 86.00 ± 4.35%, with only a marginal increase to 87.00 ± 3.61% at 48 h, confirming near-complete drug release. The results highlighted the potential of PTX-CSNPs for sustained drug delivery, with ~ 85% of PTX released within 24 h under physiological conditions (PBS pH 7.4, 37 °C). The EE% of PTX-CSNPs was 89.53 ± 1.28%, as determined by ultracentrifugation and spectrophotometric analysis of unentrapped PTX. The high EE% aligned with the near-complete drug release (87% at 48 h), indicating minimal PTX loss during preparation.

### Physical characterization

**Fig. 1:**
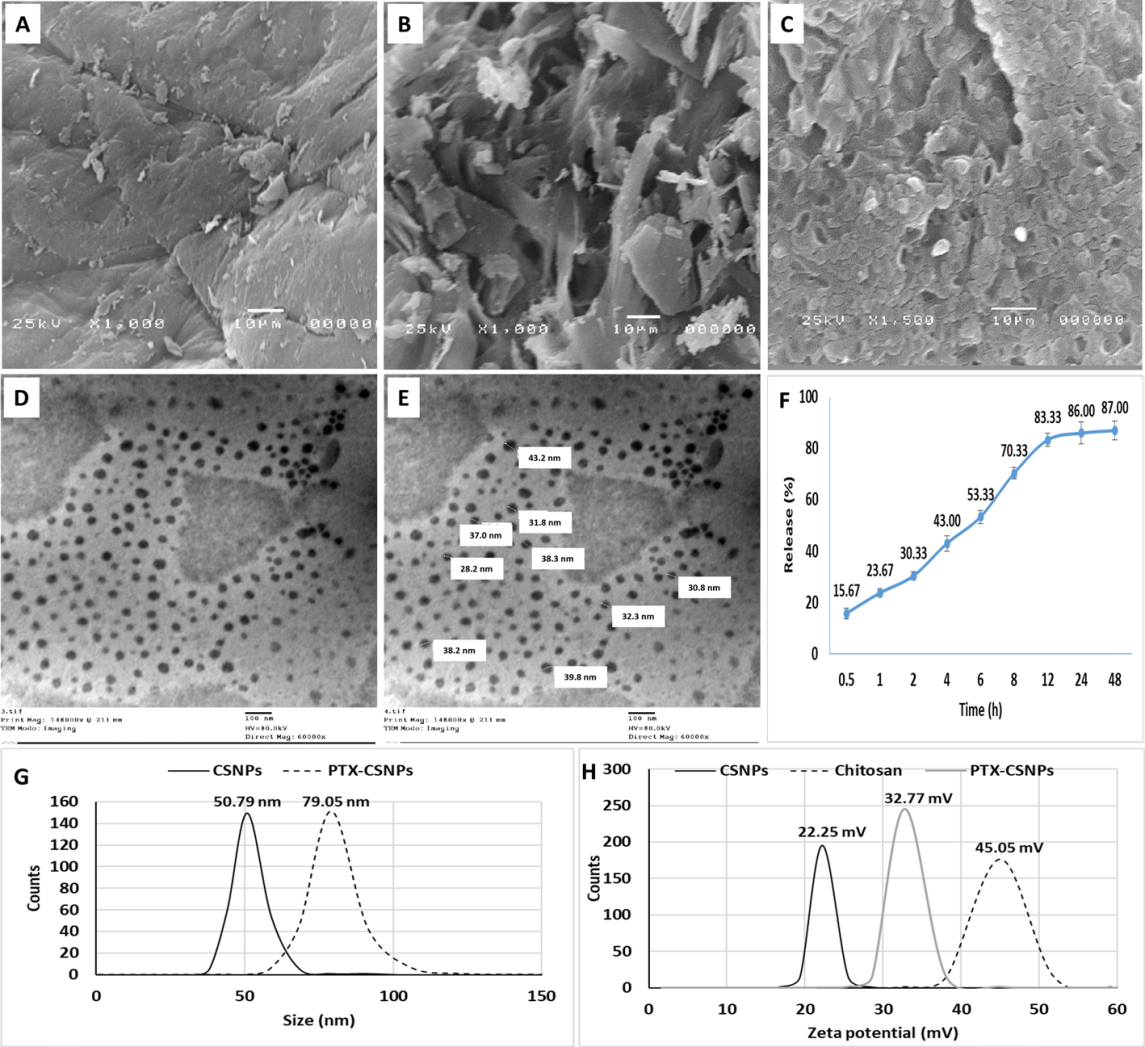
Physical characterization showing SEM images of CSNPs, PTX and PTX-CSNPs (**A**, **B** and **C**, respectively); TEM images of PTX-CSNPs (**D** and **E**); in vitro release % (**F**); size by DLS (**G**); and zeta potential (**H**)

The physical characterization of PTX-CSNPs was comprehensively analyzed using SEM and TEM (Fig. 1A–E). CSNPs are not distinctly spherical, with some clustering is evident, which is typical for chitosan-based nanoparticles due to their high surface energy and polymeric nature (Fig. 1A). Pure PTX showed a crystalline and irregular morphology. The structure appears as sharp-edged, plate-like or needle-like crystalline fragments with heterogeneous sizes. The surface is angular and well-defined, which is characteristic of crystalline drug compounds. The presence of distinct crystal facets confirms its crystalline nature prior to encapsulation (Fig. 1B). A marked morphological change was observed for PTX-CSNPs (Fig. 1C) compared to CSNPs and PTX. The nanoparticles appear more uniform and relatively spherical with a smoother and more homogeneous surface texture. The crystalline structure observed in pure PTX is no longer visible, indicating successful encapsulation of the drug within the chitosan matrix. The surface shows fewer sharp edges and a more continuous polymeric network, suggesting effective drug incorporation and nanoparticle formation. Overall, the SEM images confirm the transformation from crystalline PTX to an encapsulated form within the CSNPs, accompanied by morphological modification and improved surface uniformity.

The nanoparticles fall within the nanoscale range, visually consistent with diameters of approximately 30–50 nm. There is a relatively narrow size distribution, indicating good homogeneity in particle synthesis. The high electron density (dark appearance) of the particles suggested successful PTX loading, as heavier or denser materials absorb more electrons in TEM imaging. The surrounding lighter background indicated the presence of an amorphous or less dense matrix (chitosan polymer). Only mild aggregation is observed, with most particles appearing well-separated, implying that the formulation is likely to have good colloidal stability. DLS analysis revealed that blank CSNPs had a hydrodynamic diameter of 50.79 nm, while PTX-CSNPs exhibited a slightly larger size of 79.05 nm, indicating successful drug loading and minimal aggregation (Fig. 1G). Zeta potential measurements demonstrated that unmodified chitosan had the highest surface charge (+ 45.05 mV), followed by PTX-CSNPs (+ 32.77 mV) and blank CSNPs (+ 22.25 mV) (Fig. 1H).

The observed difference, in nanoparticle’s size, between the electron microscopy (30–50 nm) and DLS (79.05 nm) measurements is expected and aligns with established principles of nanoparticle characterization. TEM images represent the dry, core diameter of individual nanoparticles under high vacuum, excluding any surface-associated layers. In contrast, DLS measures the hydrodynamic diameter in suspension, which includes the nanoparticle core, the chitosan polymer shell, any surface-bound water molecules (hydration layer), and the contribution of the entrapped PTX. This hydrodynamic diameter is inherently larger and is influenced by solvent interactions, particle swelling, and the dynamic diffusion of particles in solution. Therefore, the DLS-reported size of ~ 79 nm, which is consistent with successful drug loading and colloidal stability, provides the relevant size for biological interactions in vitro and in vivo, while TEM/SEM confirms the nanoscale morphology and monodispersity of the prepared formulation.

### FTIR results for PTX-CSNPs

**Fig. 2:**
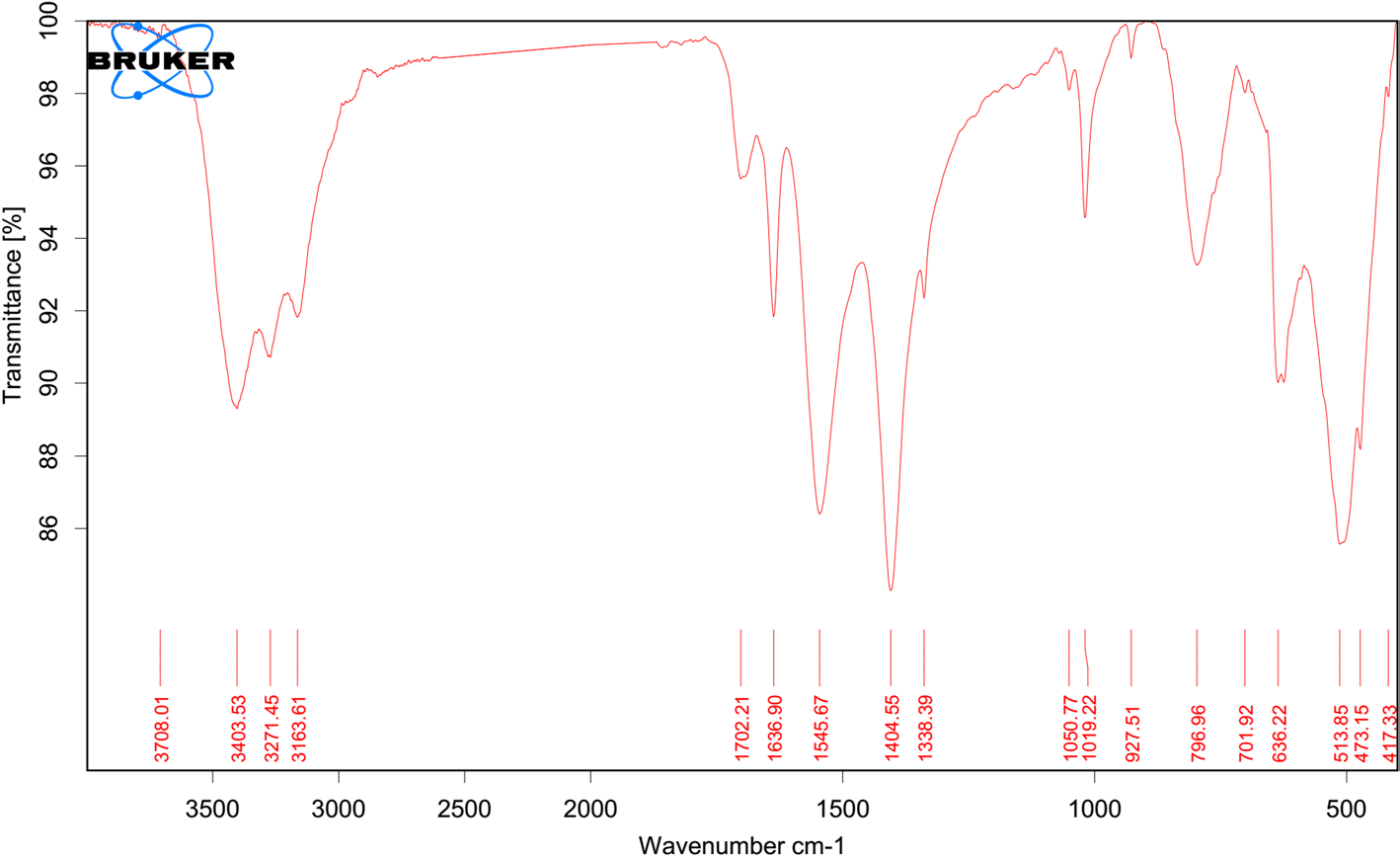
FTIR spectrum of PTX-CSNPs

The FTIR spectrum of PTX-CSNPs revealed characteristic peaks corresponding to functional groups from both chitosan and PTX, confirming successful drug encapsulation and interactions (Fig. 2). Chitosan broad peak at 3403 cm^−1^ indicated O–H and N–H stretching vibrations (hydroxyl and amine groups of chitosan). Other peaks at 1636 cm^−1^, 1545 cm^−1^ and 1050 cm^−1^ indicated C=O stretching (amide I, residual acetyl groups in chitosan), N–H bending (amide II) and C–O–C stretching (saccharide ring of chitosan). Moreover, the peaks of PTX were at 1702 cm^−1^ indicating C=O stretching (ester/carbonyl groups of PTX); 1404 cm^−1^ and 1338 cm^−1^ for C–H bending and C–N stretching (PTX alkyl/amine moieties). The loading of PTX on CSNPs was indicated from the shift in N–H/O–H peaks (3403 → 3271 cm^−1^) that suggested hydrogen bonding between PTX (C=O) and chitosan (–NH_2_/–OH). Attenuation of PTX’s 1702 cm^−1^ peak indicated reduced crystallinity due to molecular dispersion in the chitosan matrix. Absence of new peaks confirmed no covalent bonding, while peak broadening implies physical interactions (electrostatic, H-bonding).

### XRD results for PTX-CSNPs

**Fig. 3:**
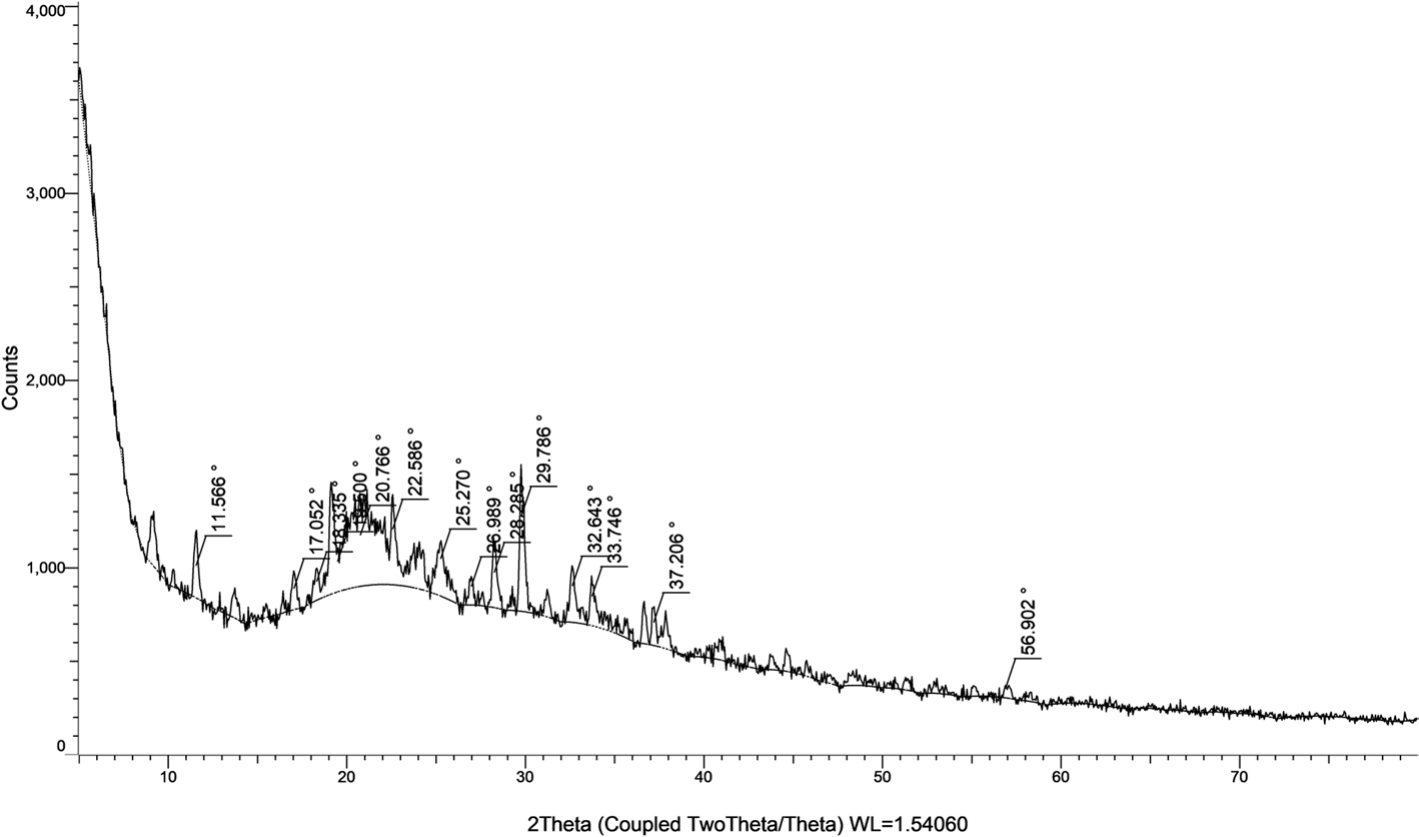
XRD pattern of PTX-CSNPs. The broad peak at 20.7° (2θ) confirms chitosan’s amorphous structure, while the absence of PTX’s crystalline peaks indicates molecular dispersion. Data collected at λ = 1.5406 Å (Cu-Kα)

The XRD pattern of PTX-CSNPs revealed key structural features, demonstrating successful drug encapsulation and changes in crystallinity (Fig. 3). The pattern showed a broad amorphous halo centered at approximately 20.7° (2θ), characteristic of chitosan’s semi-crystalline structure, while the characteristic sharp diffraction peaks of crystalline PTX (typically observed at 4.50°, 13.21°, and 15.12°) were notably absent or significantly diminished. This loss of drug crystallinity indicated molecular-level dispersion of PTX within the chitosan matrix, a key feature for enhancing drug solubility and bioavailability. The appearance of weak, broad peaks at 18.3° and 26.3° suggested some reorganization of the drug-polymer complex, likely due to intermolecular interactions between PTX and chitosan chains. These XRD results demonstrated that the ionic gelation method successfully produced nanoparticles with an amorphous drug phase dispersed within the polymer matrix, which is optimal for controlled drug release applications. The maintenance of chitosan’s broad diffraction feature confirmed the preservation of the polymer’s structural integrity during nanoparticles formation.

### In vitro characterization of PTX-CSNPs

#### Antioxidant activity (DPPH assay)

**Fig. 4:**
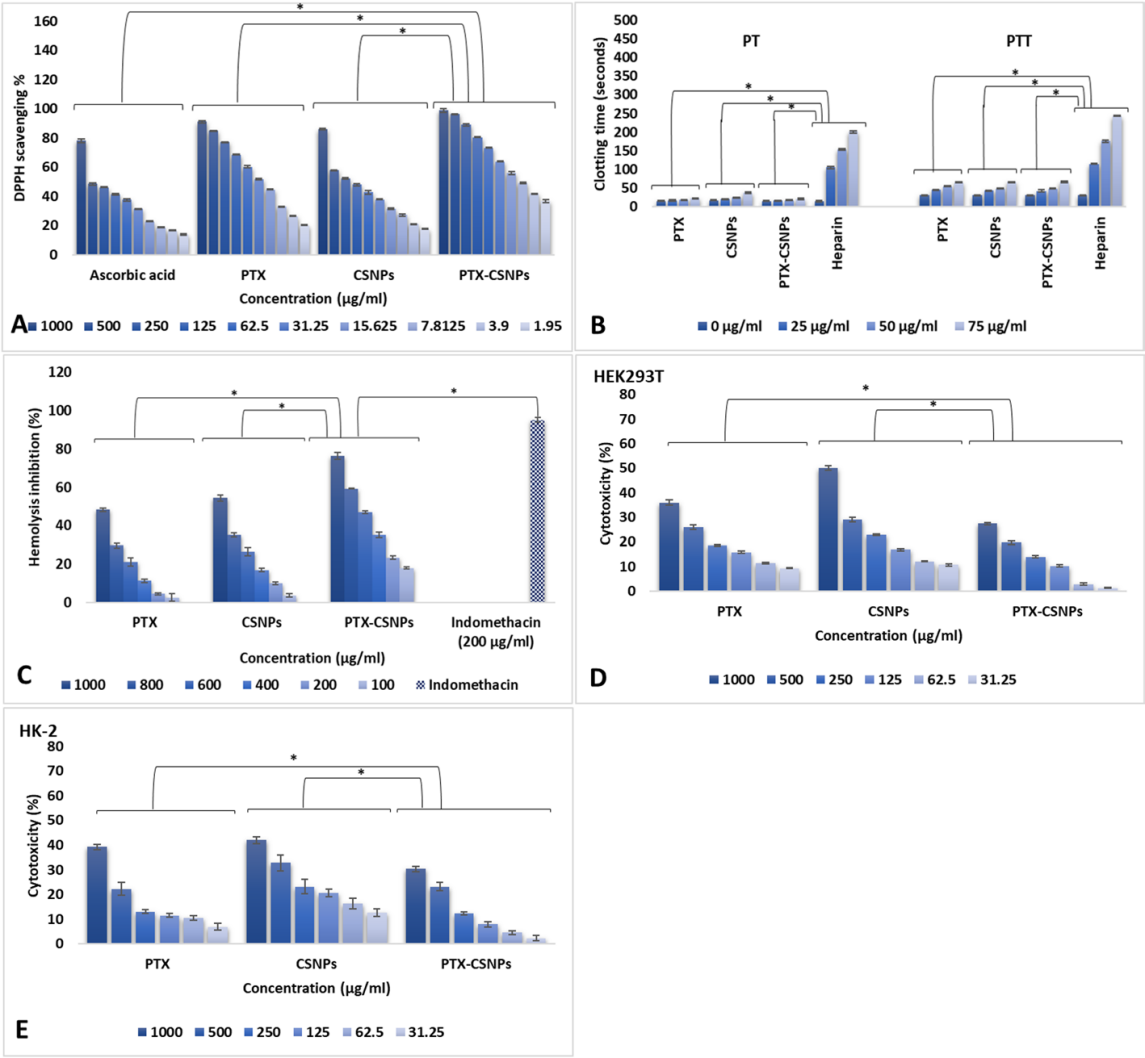
In vitro evaluation of of PTX, CSNPs and PTX-CSNPs showing antioxidant activity (DPPH scavenging capability) (**A**), anticoagulant activity (PT and PTT) (**B**), anti-inflammatory activity (hemolysis inhibition assay) (**C**), cytotoxicity activity (MTT assay) on KEK293T (**D**) and HK-2 (**E**). Data were presented as mean ± SD, significant differences were indicated by horizontal brackets, with asterisks denoting significance levels (*p* < 0.05)

The DPPH radical scavenging assay revealed concentration-dependent antioxidant activity for all tested samples (Fig. 4A). Notably, PTX-CSNPs demonstrated superior scavenging capacity compared to both free PTX and CSNPs across all concentrations (*p* < 0.05). At the highest concentration (1000 µg/mL), PTX-CSNPs showed exceptional activity (98.73% scavenging), significantly outperforming ascorbic acid (78.17%), free PTX (91.00%), and blank CSNPs (85.97%). This enhanced activity was maintained throughout the concentration range, with PTX-CSNPs showing 96.43% scavenging at 500 µg/mL and 88.87% at 250 µg/mL, compared to 84.73% and 76.90% for free PTX at these concentrations, respectively. Unloaded CSNPs exhibited moderate antioxidant activity (85.97% at 1000 µg/mL), suggesting some inherent radical scavenging capacity of chitosan. However, the significantly higher activity of PTX-CSNPs indicated that PTX encapsulation potentiated this effect. Free PTX itself showed substantial antioxidant activity (91.00% at 1000 µg/mL), but the nanoformulation consistently demonstrated superior performance at all tested concentrations. The results suggested that the CSNPs delivery system not only preserved but enhanced the antioxidant properties of PTX, likely through improved solubility and bioavailability. These findings position PTX-CSNPs as a promising therapeutic option for oxidative stress-related conditions.

#### Anticoagulant activity (PT and PTT assay)

The anticoagulant activity of PTX, CSNPs, and PTX-CSNPs was evaluated by measuring PT and PTT, with heparin serving as positive control (Fig. 4B). At baseline (0 µg/mL), all test samples showed comparable clotting times to the control (PT: ~14.33s; PTT: ~30.33s). However, heparin demonstrated a dramatic dose-dependent increase in both PT (104.33–200.33s) and PTT (114.33–243.33s) at concentrations of 25–75 µg/mL, confirming its potent anticoagulant effects. In contrast, PTX, CSNPs, and PTX-CSNPs exhibited only modest increases in clotting times. PTX-CSNPs showed slightly prolonged PT (15.93–19.63s) and PTT (41.43–65.73s) compared to baseline, but these values remained substantially lower than heparin’s effects. Unloaded CSNPs displayed the greatest anticoagulant activity among the test samples, particularly at higher concentrations (PT: 36.63s and PTT: 65.33s at 75 µg/mL), suggesting that CSNPs have inherent anticoagulant properties. Free PTX showed minimal effects on PT (16.53–21.23s) and PTT (44.13–64.43s), indicating weak anticoagulant activity. These results demonstrated that while PTX-CSNPs exhibited mild anticoagulant effects, they were significantly less potent than heparin, which may be advantageous for applications requiring minimal interference with normal coagulation. The enhanced activity of CSNPs compared to PTX alone suggested that the chitosan matrix contributed to the observed anticoagulant effects.

#### Anti-inflammatory activity (RBCs membrane stabilization assay)

The anti-inflammatory effects of PTX, CSNPs, and PTX-CSNPs were evaluated using RBCs membrane stabilization assay, with indomethacin (200 µg/mL) as positive control showing 95.26% inhibition (Fig. 4C). PTX-CSNPs demonstrated significantly superior dose-dependent protection against hypotonicity-induced hemolysis compared to both free PTX and CSNPs. At the highest concentration tested (1000 µg/mL), PTX-CSNPs exhibited 76.46% inhibition, substantially greater than free PTX (48.26%) and CSNPs (54.37%). This enhanced activity was maintained across all concentrations, with PTX-CSNPs showing 59.37% inhibition at 800 µg/mL and 47.07% at 600 µg/mL, compared to 29.63% and 21.13% for free PTX at these concentrations, respectively. CSNPs displayed moderate activity (54.37% at 1000 µg/mL), suggesting some inherent membrane-stabilizing properties of chitosan. However, the significantly higher activity of PTX-CSNPs indicated that drug encapsulation potentiated this anti-inflammatory effect. Free PTX showed the weakest activity among the tested formulations, with only 48.27% inhibition at 1000 µg/mL. The results demonstrated that nanoformulation not only preserved but enhanced the anti-inflammatory properties of PTX, likely through improved drug delivery and bioavailability, making PTX-CSNPs a promising candidate for inflammation-related therapeutic applications.

#### Cytotoxicity activity (MTT assay)

The safety profiles of PTX, CSNPs, and PTX-CSNPs were evaluated by measuring their cytotoxic effects on HEK293T and HK-2 human kidney cell lines using the MTT assay, with results expressed as percentage cytotoxicity (Fig. 4D, E). PTX-CSNPs demonstrated significantly reduced cytotoxicity compared to both free PTX and blank CSNPs across most tested concentrations in both cell lines, indicating an improved safety profile of the nanoformulation. In HEK293T cells, PTX-CSNPs exhibited the lowest cytotoxicity at all concentrations. At the highest dose (1000 µg/mL), PTX-CSNPs showed 27.40% cytotoxicity, which was substantially lower than free PTX (35.93%) and blank CSNPs (50.13%). This enhanced safety margin was particularly evident at lower cytotoxicity dropped to 2.86%, compared to 11.46% for free PTX and 12.0% for CSNPs.

A similar protective trend was observed in the more disease-relevant HK-2 human proximal tubular epithelial cells. PTX-CSNPs again showed the lowest cytotoxicity (30.33% at 1000 µg/mL), outperforming both free PTX (39.23%) and blank CSNPs (41.93%). The dose-dependent reduction in cytotoxicity was consistent, with PTX-CSNPs maintaining minimal cytotoxicity (4.53%) at 62.5 µg/mL, while free PTX and CSNPs showed 10.40% and 16.30% cytotoxicity, respectively. These results clearly demonstrate that encapsulation of PTX within chitosan nanoparticles mitigates its direct cytotoxic effects on renal cells. The superior safety profile of PTX-CSNPs, particularly in HK-2 cells directly implicated in drug-induced kidney injury, provides a strong mechanistic rationale for the enhanced renoprotection observed in vivo.

### In vivo experiment

#### Kidney function

**Fig. 5:**
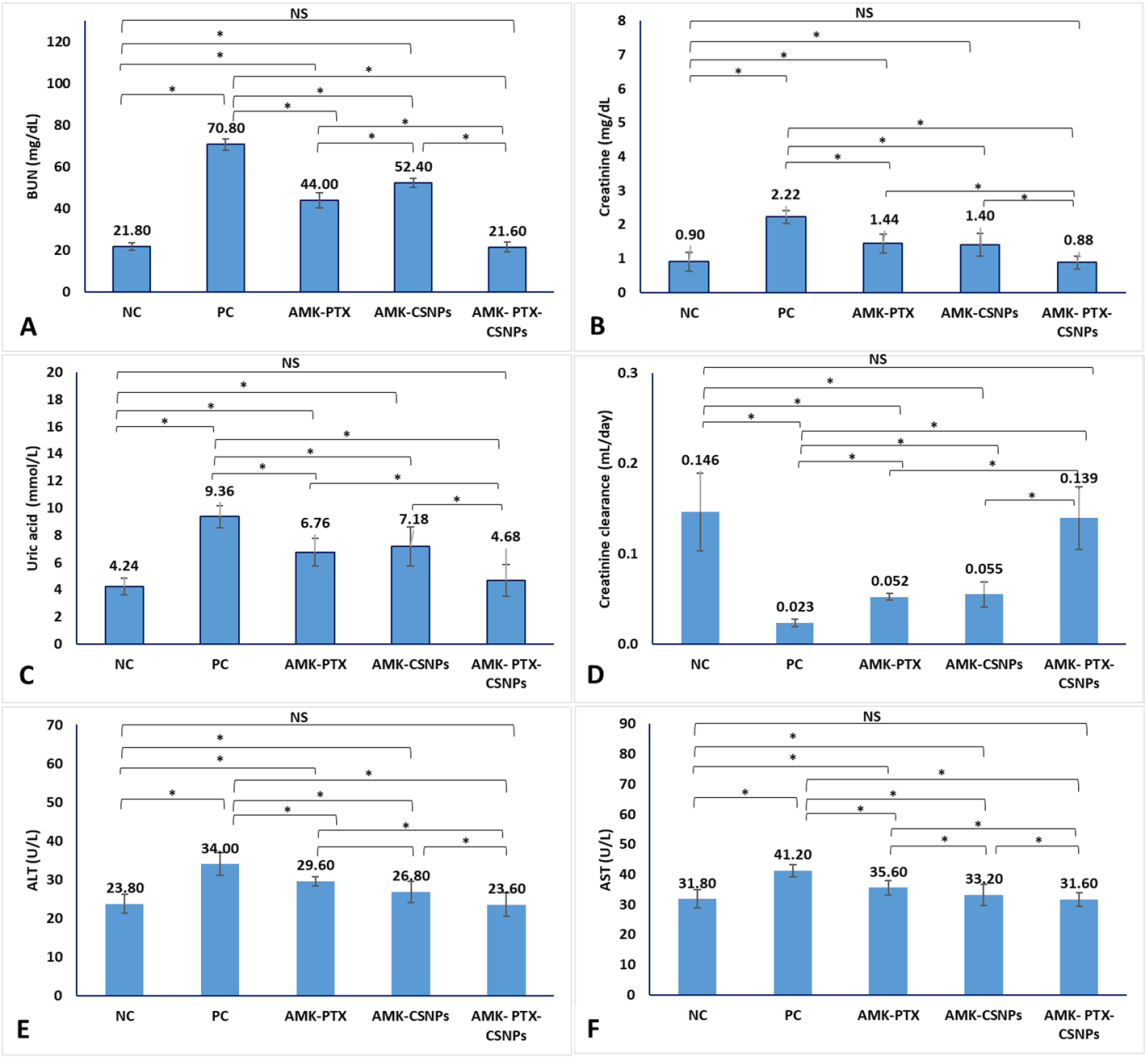
Kidney and liver function parameters in different experimental groups showing levels of BUN (**A**), creatinine (**B**), uric acid (**C**), creatinine clearance (**D**), ALT (**E**) and AST (**F**). Data were presented as mean ± SD. Statistical significance was determined by one-way ANOVA followed by Tukey’s post hoc test. Significant differences between groups are indicated by horizontal brackets, with asterisks denoting significance levels (*p* < 0.05). ns = not significant

The study evaluated the nephroprotective effects of PTX, CSNPs, and PTX-CSNPs in an amikacin-induced kidney injury model (Fig. 5). Compared to the PC group, which showed severe kidney dysfunction (BUN: 70.80 mg/dl; creatinine: 2.22 mg/dl; uric acid: 9.36 mmol/l), PTX-CSNPs demonstrated normalization to levels matching healthy controls (BUN: 21.60 mg/dl; creatinine: 0.88 mg/dl; uric acid: 4.68 mmol/l) that was matching the healthy NC group. Treatment with PTX (AMK-PTX) and unloaded CSNPs (AMK-CSNPs) provided partial protection, with PTX being slightly more effective (BUN: 44.00 vs. 52.40 mg/dl; uric acid: 6.76 vs. 7.18 mmol/l). Notably, PTX-CSNPs outperformed both free PTX and CSNPs alone, suggesting a synergistic effect of PTX and chitosan in mitigating AMK-induced nephrotoxicity. These results highlighted PTX-CSNPs as a promising therapeutic strategy for drug-induced kidney injury.

To dynamically assess glomerular filtration rate (GFR), creatinine clearance was calculated from 24-hour urine collections. Amikacin-induced nephrotoxicity resulted in a severe reduction in renal filtration function, with creatinine clearance in the PC group falling to 0.023 mL/day (Fig. 5D). This represented only ~ 16% of the NC group value (0.146 mL/day), confirming significant impairment of glomerular filtration consistent with the observed oliguria and elevated serum creatinine. Treatment with free PTX or blank CSNPs provided only partial functional recovery, increasing clearance to 0.052 mL/day and 0.055 mL/day, respectively. In contrast, PTX-CSNPs treatment effectively normalized glomerular filtration, with creatinine clearance reaching 0.139 mL/day. This value was statistically indistinguishable from healthy controls and significantly higher than all other treatment groups. These direct clearance measurements provide functional validation that PTX-CSNPs not only normalized serum biomarkers but also enhanced the fundamental filtration capacity of the kidney, offering a comprehensive demonstration of their renoprotective efficacy.

#### Liver function

To evaluate potential hepatotoxicity, serum levels of ALT and AST were measured across all experimental groups. Amikacin administration induced significant hepatotoxicity, as evidenced by elevated transaminase levels in the PC group. Serum ALT increased from 23.80 U/L in NC group to 34.00 U/L (Fig. 5E), while AST rose from 31.80 U/L to 41.20 U/L (Fig. 5F). Treatment with free PTX (AMK-PTX) partially attenuated this hepatic injury, reducing ALT to 29.60 U/L and AST to 35.60 U/L. Blank CS NPs (AMK-CSNPs) showed a similar protective trend, with ALT and AST levels of 26.80 U/L and 33.20 U/L, respectively. Notably, PTX-CSNPs treatment completely prevented amikacin-induced hepatotoxicity, restoring both ALT (23.60 U/L) and AST (31.60 U/L) to levels statistically indistinguishable from healthy controls. These findings confirm the systemic safety of PTX-CSNPs and demonstrate their additional hepatoprotective effects beyond the primary renoprotective action.

#### Oxidative stress and inflammatory markers

**Fig. 6:**
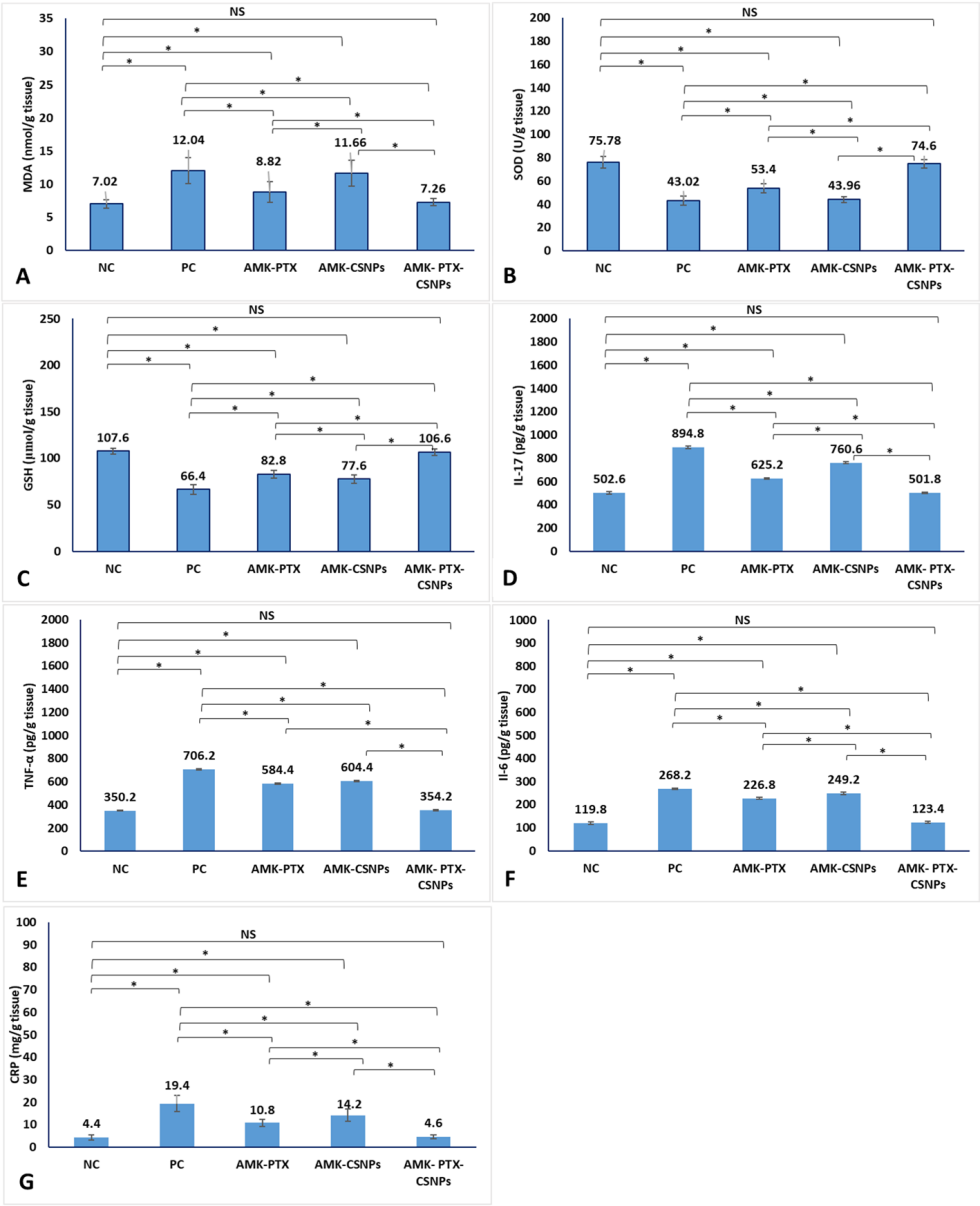
Oxidative stress and inflammatory markers in kidney tissue homogenates of different experimental groups showing levels of MDA (**A**), SOD (**B**), GSH (**C**), IL-17 (**D**), TNF-α (**E**), IL-6 (**F**) and CRP (**G**). Data are presented as mean ± SD. Statistical significance was determined by one-way ANOVA followed by Tukey’s post hoc test. Significant differences between groups are indicated by horizontal brackets, with asterisks denoting significance levels (*p* < 0.05). ns = not significant

The study revealed significant oxidative stress and systemic inflammation in PC group compared to NC group (Fig. 6). Oxidative damage was evidenced by elevated malondialdehyde (MDA: 12.04 vs. 7.02 nmol/g tissue) and markedly reduced antioxidant defenses, including SOD (43.02 vs. 75.78 U/g tissue) and GSH (66.4 vs. 107.6 µmol/g tissue). Concurrently, a pronounced inflammatory response was observed, with significant elevations in IL-17 (894.8 vs. 502.6 pg/g tissue), TNF-α (706.2 vs. 350.2 pg/g tissue), IL-6 (268.2 vs. 119.8 pg/g tissue), and CRP (19.4 vs. 4.4 mg/g tissue). Treatment with PTX-CSNPs completely reversed these pathological alterations, restoring all markers to levels statistically indistinguishable from the healthy NC group: MDA (7.26 nmol/g tissue), SOD (74.6 U/g tissue), GSH (106.6 µmol/g tissue), IL-17 (501.8 pg/g tissue), TNF-α (354.2 pg/g tissue), IL-6 (123.4 pg/g tissue), and CRP (4.6 mg/g tissue).

Free PTX treatment provided moderate protection, partially attenuating oxidative stress (MDA: 8.82 nmol/g tissue; SOD: 53.4 U/g tissue; GSH: 82.8 µmol/g tissue) and inflammation (IL-17: 625.2 pg/g tissue; TNF-α: 584.4 pg/g tissue; IL-6: 226.8 pg/g tissue; CRP: 10.8 mg/g tissue). Blank CSNPs showed minimal effects on oxidative markers (MDA: 11.66 nmol/g tissue; SOD: 43.96 U/g tissue) but exhibited some anti-inflammatory activity, particularly on IL-17 (760.6 pg/g tissue) and CRP (14.2 mg/g tissue). The superior efficacy of PTX-CSNPs in simultaneously normalizing both oxidative stress and inflammatory pathways highlights their dual therapeutic action and supports their role as a potent nanotherapeutic strategy against amikacin-induced nephrotoxicity.

#### Histopathological examination

**Fig. 7:**
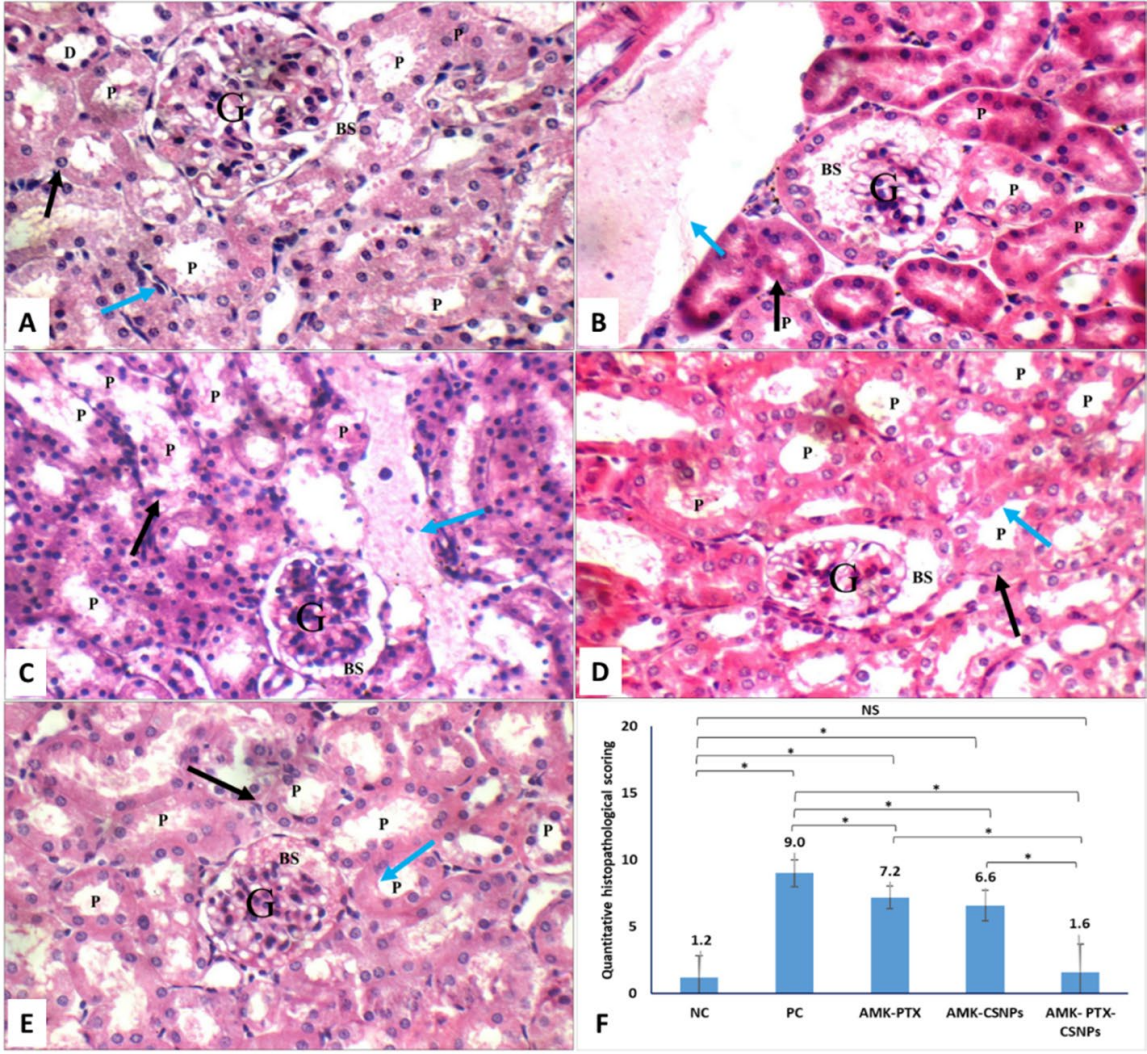
Haematoxylin and eosin kidney sections showing **A**] average glomeruli (G) with average Bowman’s spaces (BS), proximal tubules (P) with average epithelial lining (black arrow), and average interstitium (blue arrow) in healthy negative control (NC) group (X 400), **B**] atrophied glomeruli (G) with widened Bowman’s spaces (BS), proximal tubules (P) with markedly apoptotic epithelial lining (black arrow), and markedly dilated interstitial blood vessels (blue arrow) in positive control (PC) untreated group (X 400), **C**] average glomeruli (G) with average Bowman’s spaces (BS), proximal tubules (P) with scattered apoptotic epithelial lining (black arrow) and mildly dilated congested interstitial blood vessels (blue arrow) in PTX treated AMK-induced kidney injured group (X 400), **D**] atrophid glomeruli (G) with wide Bowman’s spaces (BS), proximal tubules (P) with average epithelial lining (black arrow), and preserved brush borders (blue arrow) in CSNPs treated AMK-induced kidney injured group (X 400), **E**] average glomeruli (G) with average Bowman’s spaces (BS), proximal tubules (P) with average epithelial lining (black arrow), and preserved brush borders (blue arrow) in PTX-CSNPs treated AMK-induced kidney injured group (X 400), and **F**] total histopathological injury scores (mean ± SD). Scoring assessed glomerular size, Bowman’s space dilation, tubular damage, inflammatory infiltrate, and capillary dilation (max score = 10). Significant differences between groups were indicated by horizontal brackets, with asterisks denoting significance levels (*p* < 0.05). ns = not significant

Kidney sections revealed distinct morphological changes across treatment groups (Fig. 7). The healthy NC group showed normal glomeruli with intact Bowman’s spaces, proximal tubules with typical epithelial lining, and preserved interstitium. In contrast, the amikacin-injured PC group exhibited severe damage, including glomerular atrophy, widened Bowman’s spaces, apoptotic tubular epithelium, and dilated interstitial vessels. Treatment with free PTX partially ameliorated injury, showing near-normal glomeruli but with scattered apoptotic tubules and mild vascular congestion. CSNPs improved tubular architecture (preserved brush borders) despite persistent glomerular atrophy. Notably, PTX-CSNPs demonstrated significant histological recovery, with near-normal glomeruli, intact Bowman’s spaces, healthy tubular epithelium, and preserved brush borders, closely resembling the NC group. These results indicated that PTX-CSNPs synergistically combine PTX’s nephroprotective effects with chitosan’s structural preservation capabilities, offering superior histological recovery compared to either treatment alone.

Quantitative histopathological scoring using the established criteria objectively confirmed the observed morphological changes and revealed the superior protective efficacy of PTX-CSNPs (Fig. 7F). Amikacin-induced nephrotoxicity in the PC group resulted in severe histological damage, with a total injury score of 9.0, approaching the maximum possible score of 10. This indicated widespread structural compromise across all assessed parameters: glomerular atrophy, tubular necrosis, marked inflammatory infiltration, and capillary dilation. Treatment with free PTX provided partial structural protection, significantly reducing the total histological score to 7.2. Blank CSNPs showed a comparable level of protection, yielding a score of 6.6. Notably, PTX-CSNPs treatment demonstrated near-complete histological preservation, with a total score of 1.6. This value was statistically indistinguishable from the healthy NC group and significantly superior to both free PTX and blank CSNPs treatments. The minimal injury score in the PTX-CSNPs group quantitatively validates the qualitative observation of preserved glomerular and tubular architecture; and provides robust, objective evidence of the nanoformulation’s superior renoprotective capacity at the tissue level.

**Fig. 8:**
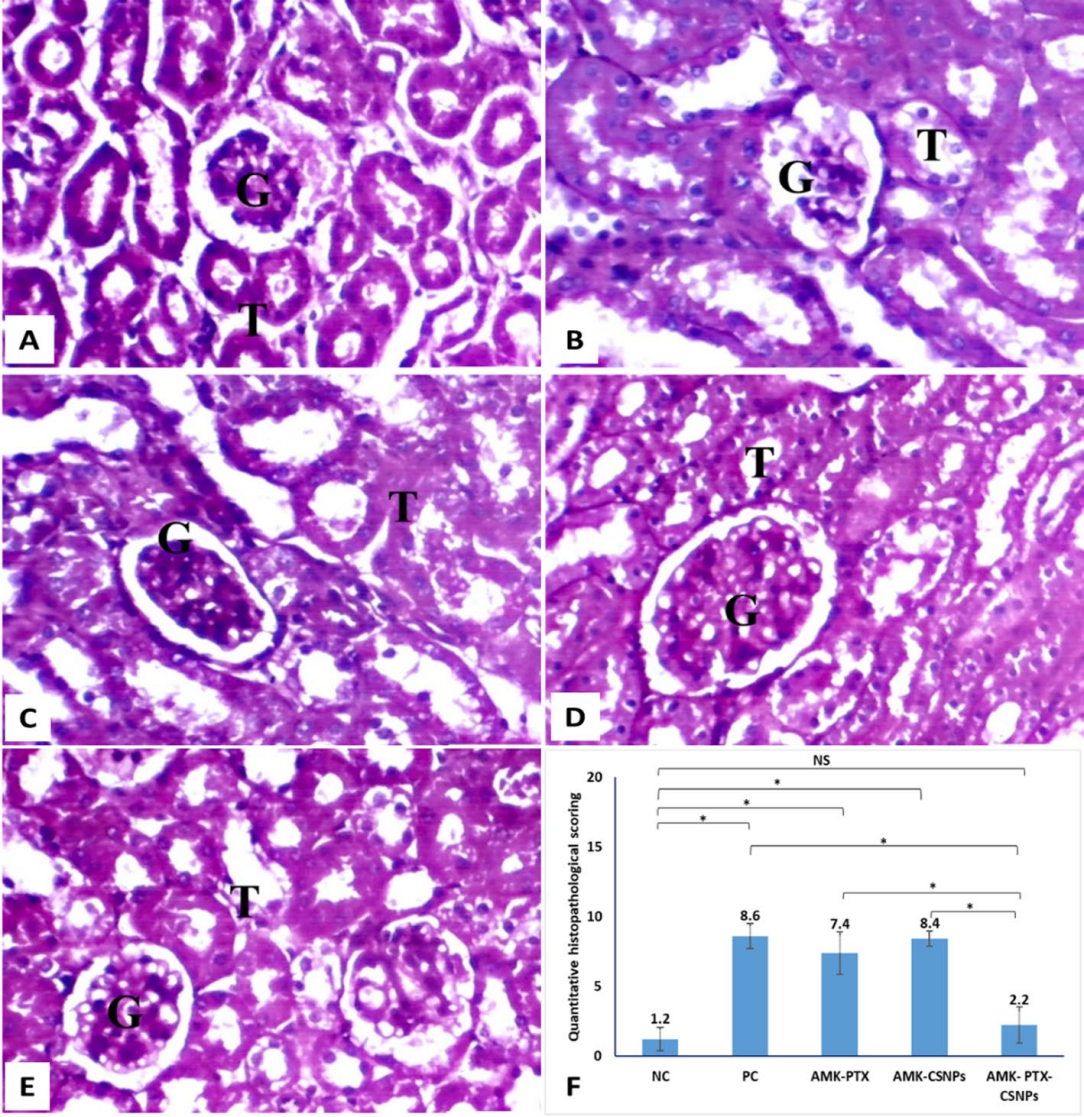
PAS-stained kidney sections showing **A**] intact glomeruli (G) with normal basement membranes and PAS-positive reaction in the glomerular and tubular basement membranes, which signifies a normal distribution of polysaccharides and glycoproteins in healthy negative control (NC) group (X 400), **B**] glomerular basement membrane thickening, loss of tubular brush borders, and prominent protein casts in positive control (PC) untreated group (X 400), **C**] partial preservation of brush borders and reduced cast formation in PTX treated AMK-induced kidney injured group (X 400), **D**] partial preservation of brush borders and reduced cast formation in CSNPs treated AMK-induced kidney injured group (X 400), **E**] near-normal PAS staining comparable to the NC group with well-preserved brush borders and minimal pathological changes in PTX-CSNPs treated AMK-induced kidney injured group (X 400), and **F**] total PAS injury scores (mean ± SD). Scoring assessed tubular brush border loss, protein cast formation, glomerular basement membrane thickening, and tubular dilatation (max score = 10). Significant differences between groups were indicated by horizontal brackets, with asterisks denoting significance levels (*p* < 0.05). ns = not significant

PAS staining of kidney sections provided enhanced visualization of carbohydrate-rich structural components, offering detailed insight into glomerular and tubular integrity across experimental groups (Fig. 8). In the NC group, PAS staining revealed intact glomeruli with normal basement membranes and strong PAS-positive reaction in both glomerular and tubular basement membranes, indicative of normal polysaccharide and glycoprotein distribution. Proximal tubules exhibited well-preserved, continuous brush borders, reflecting intact ultrastructural integrity. In contrast, the PC group displayed severe pathological alterations, including glomerular basement membrane thickening, diffuse loss of tubular brush borders, and prominent protein casts within tubular lumens. These findings are characteristic of advanced proximal tubular injury and impaired reabsorptive function. Treatment with free PTX and blank CSNPs resulted in partial morphological preservation, with moderate brush border continuity and reduced cast formation. Notably, PTX-CSNPs treatment demonstrated near-complete structural preservation, with well-maintained brush borders, minimal protein casts, and normal glomerular architecture, closely resembling the NC group.

Semiquantitative scoring of PAS-stained sections objectively confirmed these observations. The PC group exhibited a high total PAS injury score (8.6), reflecting severe structural compromise. While free PTX and blank CSNPs provided limited improvement (scores: 7.4 and 8.4, respectively), PTX-CSNPs treatment markedly reduced the injury score to 2.2; a value statistically indistinguishable from healthy controls (1.2). These findings provide stain-specific, quantitative validation of the nanoformulation’s superior capacity to preserve renal ultrastructure against amikacin-induced damage.

## Discussion

The development of PTX-CSNPs represents a promising strategy to enhance the therapeutic potential of PTX while mitigating its limitations. This study demonstrated that nanoformulation significantly improved PTX’s antioxidant, anti-inflammatory, and nephroprotective properties, while also optimizing its release kinetics and bioavailability. The findings aligned with emerging research on chitosan-based drug delivery systems, reinforcing their potential for targeted and sustained therapeutic applications.

In this study, the observed biphasic release profile of PTX from PTX-CSNPs aligned well with previous reports on chitosan-based drug delivery systems. The initial burst release (15.67% at 0.5 h; 30.33% at 2 h) was consistent with the findings of Samy et al. [46], who reported a 20–35% burst release of 5-fluorouracil from chitosan nanoparticles within 2 h, attributed to surface-adsorbed drug molecules. The release of PTX from PTX-CSNPs took place through many mechanisms like surface erosion, diffusion, disintegration or desorption [47]. The subsequent sustained release phase (83.33% at 12 h; 87% at 48 h) mirrored results from Nair et al. [48], where chitosan nanoparticles released ~ 41% of encapsulated curcumin over 24 h due to progressive matrix erosion. Dehghani et al. [38] showed that high concentration of chitosan led to slow release of PTX from pentoxifylline loaded lecithin/chitosan nanoparticles; and added that a burst release was observed with nanoparticles with low concentration of chitosan and lecithin. Moreira et al. [43] showed that PTX was released from chitosan film in two phases, fast release at 2 h and slow release at more than 2 h, with a ~ 88% release after 72 h. Notably, the near-complete release (87% at 48 h) with minimal drug loss suggested superior formulation stability compared to Moreira et al. [43], where chitosan NPs retained ~ 12% unreleased drug. These comparisons underscored the efficacy of PTX-CSNPs for sustained delivery, combining high drug loading with controlled release kinetics. The high EE% (89.53 ± 1.28%) corroborated with Dehghani et al. [38], who achieved 30–65% EE for PTX in lecithin/chitosan nanoparticles.

The increase in hydrodynamic size from 50.79 nm (CSNPs) to 79.05 nm (PTX-CSNPs) is consistent with studies on drug-loaded chitosan nanoparticles, such as Nair et al. [48], who reported a size expansion after curcumin encapsulation. Moreover, other studies reported the increase in size of CSNPs after plant extract encapsulation [49, 50]. The reduction in zeta potential from + 45.05 mV (chitosan) to + 32.77 mV (PTX-CSNPs) suggested partial shielding of chitosan’s protonated amine groups (–NH_3_^+^) by PTX, as observed by Chen et al. [51] for other hydrophobic drugs. Despite this decrease, the + 32.77 mV value of PTX-CSNPs still exceeds the ± 30 mV threshold for colloidal stability, as established by Honary and Zahir [52]. The lower zeta potential of blank CSNPs (+ 22.25 mV) compared to unmodified chitosan (+ 45.05 mV) may reflect residual TPP anions from crosslinking, which neutralize some surface charges. This aligned with Sadeghi et al. [53], who noted similar effects in TPP-crosslinked chitosan systems (+ 24.2 and 15.9 nm for chitosan and CSNPs, respectively).

The FTIR spectrum of PTX-CSNPs demonstrated successful drug encapsulation through distinct vibrational signatures and intermolecular interactions, consistent with established literature on chitosan-based drug delivery systems. The broad peak at 3403 cm^−1^ (O–H/N–H stretching) shifted to 3271 cm^−1^ in PTX-CSNPs, indicating hydrogen bonding between PTX’s carbonyl groups (C=O at 1702 cm^−1^) and chitosan’s amine groups (–NH_2_). This aligned with observations by Agnihotri et al. [54], who reported similar shifts in chitosan nanoparticles due to drug-polymer H-bonding. The attenuation of PTX’s C=O peak (1702 cm^−1^) suggests reduced drug crystallinity, mirroring findings by Nair et al. [48] for chitosan-curcumin nanoparticles, where peak broadening confirmed molecular dispersion. The preservation of chitosan’s amide I (1636 cm^−1^) and C–O–C (1050 cm^−1^) peaks confirmed structural integrity post-encapsulation. No new peaks were observed, ruling out covalent modifications. This agreed with Chen et al. [51], who noted that chitosan-drug interactions are typically physical.

The XRD analysis of PTX-CSNPs revealed significant changes in crystallinity that confirmed the successful drug encapsulation and nanoparticles formation. The characteristic broad peak observed at ~ 20.7° (2θ) corresponds to the amorphous nature of chitosan, consistent with previous reports on chitosan-based nanoparticles [55]. Notably, the sharp diffraction peaks of pure PTX (typically appearing at 4.50°, 13.21°, 15.12°) [56] were either absent or substantially reduced in intensity, indicating the transformation of crystalline PTX into an amorphous state when incorporated into the chitosan matrix [43]. This loss of drug crystallinity is particularly advantageous as it enhanced the solubility and bioavailability of the encapsulated drug [57]. The appearance of weak, broad peaks at 18.3° and 26.3° suggested some degree of molecular reorganization, likely resulting from interactions between PTX and chitosan polymer chains during nanoparticles formation [43]. These findings collectively demonstrated that the ionic gelation method effectively produced nanoparticles where the drug was molecularly dispersed within the polymer matrix, while maintaining the structural integrity of chitosan [58]. The amorphous nature of the drug-polymer complex, as evidenced by the XRD pattern, is ideal for controlled drug delivery applications, as it facilitated more uniform drug release kinetics compared to crystalline formulations [59].

PTX is a methylxanthine derivative with well-documented pharmacological effects that are significantly enhanced when encapsulated in CSNPs. In its free form, PTX exhibited moderate antioxidant activity, due to its ability to inhibit phosphodiesterase and reduce oxidative stress [60, 61]. However, when formulated as PTX-CSNPs, the antioxidant capacity increased dramatically to 98.73% scavenging at the same concentration. This enhancement aligns with findings by Di Santo et al. [62], who reported that chitosan nanoparticles can potentiate drug antioxidant effects through improved solubility and sustained release mechanisms. PTX has been known for its anti-inflammatory activity [63, 64]. The anti-inflammatory properties of PTX are also markedly improved through nanoformulation. While free PTX shows approximately 48.26% inhibition of RBCs hemolysis at 1000 µg/mL, PTX-CSNPs achieve 76.46% inhibition at the same concentration. This improvement is consistent with the work of Jafernik et al. [65], who demonstrated that chitosan nanoparticles enhance drug bioavailability and membrane stabilization. In anticoagulant assays, free PTX exhibited minimal effects on clotting times, while PTX-CSNPs show slightly prolonged activity, likely due to chitosan’s inherent interactions with clotting factors, as described by Gheorghiță et al. [66]. Cytotoxicity studies revealed that PTX-CSNPs demonstrated enhanced therapeutic potential compared to free PTX in HEK293T cells and HK-2 cells. This improvement in cytotoxic efficacy was attributed to chitosan-mediated cellular uptake [67]. These comparative results demonstrated that PTX-CSNPs not only preserved but significantly enhanced the therapeutic profile of PTX through multiple mechanisms: 1- improved drug solubility and stability, 2- enhanced cellular uptake and bioavailability, and 3- synergistic combination of PTX’s pharmacological effects with chitosan’s inherent biological activities. The multifunctional capabilities of PTX-CSNPs make them a promising candidate for treating conditions involving oxidative stress, inflammation, and cellular proliferation, while potentially reducing the dose requirements and side effects associated with conventional PTX therapy.

The favorable in vitro safety profile of PTX-CSNPs (IC₅₀ > 1000 µg/mL) is consistent with the well-tolerated in vivo dose of 50 mg/kg. While the administered dose appears numerically higher than tested in vitro concentrations, pharmacokinetic principles of distribution and clearance reconcile this apparent discrepancy. The total administered dose distributes into the significant volume of total body water and tissues, undergoes metabolism and excretion, resulting in far lower steady-state plasma and renal tissue concentrations [68–70]. Simple dilutional modeling confirms that even a worst-case estimate of immediate plasma distribution yields concentrations within the safe in vitro range, supporting the translational relevance and safety of the selected dosing regimen.

The study demonstrated that PTX-CSNPs offered superior protection against AMK-induced nephrotoxicity compared to free PTX or unloaded CSNPs. Biochemically, PTX-CSNPs restored kidney function markers (BUN, creatinine, and uric acid) to near-normal levels, matching healthy controls, while free PTX and CSNPs provided only partial protection. The normalization of oxidative stress markers (MDA, SOD, and GSH) and inflammatory markers (IL-17, TNF-α, IL-6, and CRP) by PTX-CSNPs suggested a synergistic mechanism: chitosan’s intrinsic antioxidant properties combined with PTX’s ability to inhibit TNF-α and oxidative pathways. Histopathological findings further validated this synergy, with PTX-CSNPs preserving glomerular and tubular architecture, while free PTX showed residual tubular apoptosis. Notably, CSNPs improved tubular brush borders, consistent with chitosan’s documented role in epithelial repair. The dual action of PTX-CSNPs, simultaneously mitigating oxidative damage and inflammation, mirrors trends observed in other drug-loaded chitosan systems [71]. These results position PTX-CSNPs as a promising strategy to counteract antibiotic-induced kidney injury, addressing both functional and structural damage. The superior restoration of renal function, oxidative balance, and histoarchitecture in the PTX-CSNPs group, despite administration of the same PTX dose as the free drug group, suggests enhanced renal drug availability facilitated by the chitosan nanocarrier.

In this study, the assessment of hepatic transaminases revealed that amikacin administration induced a significant but mild hepatotoxic effect, elevating both ALT and AST levels in the PC group. This finding aligns with clinical and preclinical reports indicating that aminoglycosides, while primarily nephro- and ototoxic, can also affect hepatic function through oxidative stress and mitochondrial dysfunction in hepatocytes [16, 56]. The observed increase (in ALT and AST) is consistent with the moderate, dose-dependent hepatotoxicity documented in rodent models of aminoglycoside exposure.

Importantly, PTX-CSNPs treatment completely prevented this hepatic enzyme elevation, restoring ALT and AST to baseline levels. This hepatoprotective effect can be attributed to several synergistic mechanisms: 1- PTX’s known anti-inflammatory and antioxidant properties; as it inhibits TNF-α and reduces oxidative stress in hepatic tissues, as demonstrated in models of drug-induced liver injury [72, 73], and 2- CS NPs have been shown to ameliorate hepatotoxicity by scavenging free radicals, reducing lipid peroxidation, and enhancing endogenous antioxidant defenses [74]. The partial protection offered by free PTX and blank CSNPs further supports a multifactorial mechanism, where both the drug and the nanocarrier contribute to hepatic safety. Notably, the complete normalization achieved only with PTX-CSNPs underscores the formulation’s superiority, likely due to improved bioavailability and sustained release of PTX, coupled with chitosan’s intrinsic bioactivity. These results expand the therapeutic promise of PTX-CSNPs beyond renoprotection to include systemic safety and multi-organ protective effects. This is particularly relevant for clinical translation, as patients receiving aminoglycosides often present with comorbidities affecting both renal and hepatic function.

Extensive research has demonstrated the therapeutic potential of various pharmacological agents in mitigating free radical-mediated tissue damage, promoting tissue regeneration, and improving patient outcomes. Among these, PTX, a methylxanthine derivative with vasodilatory properties, has emerged as a particularly promising therapeutic agent. Originally developed for treating peripheral vascular diseases [75], PTX has shown remarkable pleiotropic effects that extend beyond its primary indication. As highlighted by Hassan et al. [76], PTX exerts significant immunomodulatory effects by regulating key cytokines including TNF-α, IL-1β, IL-6, and TGF-β1, which play crucial roles in tissue repair and extracellular matrix remodelling.

The antioxidant properties of PTX have been extensively documented across multiple experimental models. Studies by Eğin et al. [72] and Sunil et al. [73] demonstrated PTX’s ability to reduce reactive oxygen species (ROS) production while enhancing endogenous antioxidant defenses, including SOD and catalase (CAT) activities. These findings are further supported by Dhulqarnain et al. [77], who reported that PTX modulates oxidative stress-related gene expression, providing mechanistic insights into its tissue-protective effects. Comparative studies have established PTX’s superior antioxidant efficacy relative to other agents such as quinacrine and vitamin E [64, 78], particularly in preventing lipid peroxidation and maintaining cellular redox balance.

The current study builds upon this substantial body of evidence by demonstrating that PTX’s therapeutic potential can be significantly enhanced through nanoformulation. While previous research by Radfar et al. [79] and Dinckan et al. [80] established PTX’s efficacy in reducing oxidative stress in clinical settings, our findings revealed that chitosan nanoparticle encapsulation amplifies these benefits through improved drug delivery and bioavailability. This is particularly evident in the restoration of renal antioxidant enzymes and inflammatory markers to baseline levels, outperforming both free PTX and CSNPs. The observed nephroprotection aligns with Nesek-Adam et al.‘s [81] work on PTX’s organ-protective effects while overcoming the pharmacokinetic limitations noted in earlier studies.

The unique properties of CSNPs make them ideal candidates for developing stimuli-responsive nanocarriers in targeted therapy. As demonstrated by Su and Kang [82], CSNPs can be engineered to deliver chemotherapeutic drugs specifically to sites while minimizing toxicity to healthy cells through pH-responsive drug release mechanisms. This targeted delivery capability is particularly valuable given chitosan’s excellent biocompatibility, biodegradability, and low toxicity profile [83]. The versatility of CSNPs is further evidenced by their adaptability to various administration routes including oral, intravenous, and transdermal delivery systems. However, a critical challenge in systemic applications involves the rapid clearance of CSNPs by the reticuloendothelial system (RES), as noted by Ghassemi et al. [84]. This instability in circulation can be mitigated through surface modifications such as PEGylation or ligand conjugation, which have been shown to enhance circulation time. Importantly, numerous studies including Herdiana et al. [71] have documented CSNPs’ ability to significantly improve the solubility, stability, and bioavailability of hydrophobic drugs like paclitaxel and docetaxel through molecular encapsulation and controlled release mechanisms.

The superior renoprotective efficacy of PTX-CSNPs, despite administration of an equivalent PTX dose, strongly suggests enhanced drug delivery to renal tissue. This is mechanistically supported by two interrelated physicochemical properties of the nanoformulation. 1- surface charge-driven interaction: The positive zeta potential of PTX-CSNPs (+ 32.77 mV) promotes electrostatic binding to negatively charged components of the glomerular filtration barrier, including heparan sulfate proteoglycans in the glomerular basement membrane and the glycocalyx of proximal tubular cells, thereby prolonging renal retention. Cationic macromolecules and nanoparticles are known to exhibit greater renal accumulation than anionic or neutral counterparts due to this charge affinity [85–87]. Furthermore, amphiphilic chitosan derivatives have been shown to interact with organic cation transporter 2 (OCT2) expressed on the basolateral membrane of proximal tubular epithelial cells, reducing clearance of cationic substrates and enhancing tubular uptake [88]. 2- size-dependent passive targeting: With a hydrodynamic diameter of ~ 79 nm, PTX-CSNPs fall within the optimal size window (20–100 nm) for passive renal targeting. Nanoparticles smaller than 6–8 nm undergo rapid glomerular filtration and urinary excretion, whereas those exceeding 200 nm are predominantly cleared by the reticuloendothelial system (liver and spleen). Importantly, in the context of acute kidney injury, the compromised glomerular filtration barrier exhibits increased permeability, leading to 1.9-fold greater accumulation of sub-100 nm nanoparticles in diseased glomeruli compared to healthy controls [89]. This “passive targeting” effect, driven by disease pathophysiology, likely contributed to the preferential renal deposition of PTX-CSNPs in our amikacin-injured model. Finally, emerging evidence indicates that chitosan-based carriers (l-serine–modified chitosan) can exploit kidney injury molecule-1 (KIM1), a phosphatidylserine receptor markedly upregulated on the apical membrane of injured proximal tubular epithelial cells [90], to achieve active, injury-selective drug delivery [91]. Collectively, these charge-, size-, and injury-dependent mechanisms provide a robust mechanistic framework for the enhanced renal availability and therapeutic superiority of PTX-CSNPs observed in this study.

Recent studies have demonstrated the successful incorporation of PTX into nanoparticle systems to enhance its therapeutic efficacy. Moreira et al. [43] developed PTX-loaded chitosan films for wound healing, achieving a high drug loading efficiency and sustained release over 72 h, which significantly improved wound contraction (by day 14) and reduced inflammation (decrease in TNF-α levels) compared to free PTX. Similarly, Rençber et al. [92] formulated mucoadhesive PTX-loaded Eudragit nanoparticles for vaginal delivery, optimizing particle characteristics (180 nm size, + 28 mV zeta potential) to enhance mucosal adhesion and pH-responsive drug release. Their results showed 3-fold higher drug penetration and significant ulcer healing in 80% of animal models, with no epithelial irritation. These studies highlighted the versatility of PTX nanoformulations in addressing diverse medical conditions through improved drug delivery, sustained release, and targeted action. Our findings with PTX-CSNPs aligned with these advancements, demonstrating superior nephroprotection, antioxidant effects, and anti-inflammatory properties compared to free PTX. The success of these formulations underscored the potential of nanotechnology to overcome PTX’s limitations, such as poor bioavailability and rapid clearance, while enhancing its therapeutic benefits across various applications.

In conclusion, PTX-CSNPs exhibited superior therapeutic efficacy compared to free PTX, offering enhanced renal protection, antioxidant activity, and anti-inflammatory effects. The nanoformulation successfully addresses key challenges associated with PTX, including poor solubility and rapid clearance, while maintaining biocompatibility and controlled release. These results highlighted PTX-CSNPs as a viable nanotherapeutic option for conditions involving oxidative stress and inflammation, particularly in drug-induced nephrotoxicity.

This study has several limitations that should be acknowledged. First, the research was conducted primarily in animal models, which may not fully replicate human physiological responses and disease progression. Second, while we demonstrated significant renoprotective effects after oral administration of PTX-CSNPs, we did not measure renal tissue drug concentrations or perform pharmacokinetic analyses to confirm targeted renal accumulation and bioavailability. Third, the mechanistic insights into PTX-CSNPs’ interactions with renal cells at the molecular level remain incomplete. Fourth, the long-term stability and storage conditions of PTX-CSNPs were not evaluated, which is critical for practical formulation development. Finally, our therapeutic evaluation was conducted over 28 days in an acute kidney injury model; the long-term safety, potential nanoparticle accumulation, and efficacy of PTX-CSNPs in chronic kidney injury settings remain to be investigated.

Future studies should focus on: (1) conducting comprehensive pharmacokinetic and biodistribution studies to quantify renal targeting efficiency of orally administered PTX-CSNPs; (2) evaluating the long-term (e.g., 3–6 month) safety and therapeutic efficacy of PTX-CSNPs in chronic kidney disease models; (3) exploring surface modifications such as PEGylation to enhance systemic circulation time and renal accumulation; and (4) investigating molecular mechanisms of action to further elucidate the renoprotective pathways. Addressing these gaps will strengthen the translational potential of PTX-CSNPs as a promising nanotherapeutic for drug-induced and chronic kidney injury.

## Data Availability

All data generated or analysed during this study are available from the corresponding author on a reasonable request.

## Abbreviations

AMK: Amikacin
BUN: Blood urea nitrogen
CKD: Chronic kidney disease
CNS: Central nervous system
CSNPs: Chitosan nanoparticles
DPPH: 1,1-Diphenyl-2-picrylhydrazyl (antioxidant assay)
EE%: Entrapment efficiency
FTIR: Fourier-transform infrared spectroscopy
GFR: Glomerular filtration rate
IL-1β/6/17: Interleukin-1β/6/17
MDA: Malondialdehyde (oxidative stress marker)
MTT: 3-(4,5-Dimethylthiazol-2-yl)-2,5-diphenyltetrazolium bromide (cytotoxicity assay)
NAC: N-Acetylcysteine
NPs: Nanoparticles
PBS: Phosphate-buffered saline
PT: Prothrombin time
PTT: Partial thromboplastin time
PTX: Pentoxifylline
RBCs: Red blood cells
RES: Reticuloendothelial system
ROS: Reactive oxygen species
SEM: Scanning electron microscopy
SOD: Superoxide dismutase
TEM: Transmission electron microscopy
TNF-α: Tumor necrosis factor-alpha
TPP: Sodium tripolyphosphate (crosslinker for chitosan)
XRD: X-ray diffraction
DLS: Dynamic light scattering (particle size analysis)
HBSS: Hank’s balanced salt solution
IVCs: Individually ventilated cages (animal housing)
NC: Negative control (healthy mice)
PC: Positive control (AMK-induced injury, untreated)
AMK-PTX: AMK-injured + free pentoxifylline
AMK-CSNPs: AMK-injured + blank chitosan nanoparticles
AMK-PTX-CSNPs: AMK-injured + PTX-loaded chitosan nanoparticles

## Declarations

### Generative AI and AI-assisted technologies

During the preparation of this work, the author(s) used Grammarly to improve language clarity and correct grammatical errors. After using this tool, the authors thoroughly reviewed and edited the content as needed and took full responsibility for the publication’s content.
